# Spotlight on Human Papillomavirus Vaccination Coverage: Is Nigeria Making Any Progress?

**DOI:** 10.1200/GO.23.00088

**Published:** 2023-06-15

**Authors:** Elvis Anyaehiechukwu Okolie, Blessing Ifeoma Nwadike

**Affiliations:** ^1^ School of Health and Life Sciences, Teesside University, Middlesbrough, United Kingdom; ^2^ Department of Public Health, David Umahi Federal University of Health Sciences, Uburu, Nigeria; ^3^ Department of Microbiology, University of Ibadan, Ibadan, Nigeria

The tragedy of losing one woman every 2 minutes to cervical cancer is regrettable in the face of advancements in cervical cancer prevention and treatment.^1^ Despite being a largely preventable disease through human papillomavirus (HPV) vaccination and screening, the burden of cervical cancer is increasing each year with a concomitant widening of global health inequities.^1,2^ In terms of global cancer incidence and mortality, cervical cancer ranks fourth with an estimated 604,000 new patients and 342,000 deaths in 2020.^2^ It is alarming to note that about 90% of the global cervical cancer incidence and mortality occur in low- and middle-income countries (LMICs), with women of low socioeconomic status bearing the greatest burden.^2,3^ Such disparities in cervical cancer burden demonstrate huge socioeconomic gaps between countries and inequitable implementation of cervical cancer prevention and control measures including HPV vaccination and organized screening programmes.^4^ Hence, it becomes morally imperative to address stark inequities in global cervical cancer burden and prevention efforts especially in LMICs.

In Nigeria, an estimated incidence of 12,100 patients and 8,000 deaths in 2020 puts cervical cancer as the second leading cause of female cancer morbidity and mortality behind breast cancer.^2^ Even more disturbing is that an estimated 60.9 million Nigerian women age 15 years and older will be at greater risk of developing cervical cancer in the absence of cost-effective strategies to scale up prevention and early detection.^5^ The 90-70-90 targets to be met by 2030 as contained in the global strategy to accelerate the elimination of cervical cancer as a public health problem raises optimism among LMICs including Nigeria despite skeptics describing it as overly ambitious and not realistic. Specifically, the 90-70-90 targets set out to see that 90% of girls are fully vaccinated with HPV vaccine by age 15 years, 70% of women are screened with a high-performance test at 35 years and again at 45 years and that 90% of women identified with cervical disease receive treatment.^1^

Vaccination of adolescent girls with HPV vaccines before the onset of sexual activity is the most effective long-term intervention for reducing the risk of developing cervical cancer. Knowledge of the causal relationship between HPV infection and cervical cancer has stimulated advances in the development, licensing, and usage of HPV vaccines.^6,7^ Evidence from population studies including post-vaccination follow-up has shown HPV vaccines to be highly immunogenic, safe, and effective in preventing HPV infection, cervical lesions, anogenital warts, and other HPV-related diseases.^8^ Currently, six HPV vaccines are licensed for use by the WHO—bivalent HPV vaccines (cervarix, cecolin, and walrinvax), quadrivalent HPV vaccines (gardasil and cervarax), and nonavalent HPV vaccine (gardasil9).^7^ Providing protection against high-risk (oncogenic) HPV types 16 and 18 that cause the majority of precancers, cervical cancers, and other HPV-related cancers is a unique characteristic across the licensed vaccines. WHO's guidelines recommend that adolescent girls between 9 and 14 years receive two doses of vaccine to be fully immunized with at least a 6-month interval between doses, with exceptions for special populations including immunocompromized individuals and those age 15 years or older, for whom three doses are recommended.^7^ In 2022, following evidence that a single dose of HPV vaccine provided similar protection against HPV infection as seen in multidose regimen,^9^ the WHO updated its HPV vaccination recommendations to include a single-dose schedule (also referred to as alternative or off-label single-dose schedule) for individuals age 9-20 years.^7^ The single-dose schedule update is timely and can help countries such as Nigeria to introduce the HPV vaccine, improve coverage, and cut associated costs.

As of June 2020, the number of WHO member states considered to have partially or fully introduced the HPV vaccine nationally stood at 107 (55%) of 194.^10^ Although more than 80% of high-income countries (HICs) have introduced HPV vaccines, LMICs still lag behind and have introduced HPV vaccines at a slower pace as only 41% achieved introduction as of 2019.^10^ Regrettably, 70% of girls worldwide reside in countries that are yet to nationally introduce HPV vaccines including Nigeria.^10^ From 2006 when the first HPV vaccine was licensed to 2017, more than 100 million girls globally have been reached with at least one dose of HPV vaccine, and it is worrisome that 95% of vaccinated girls were resident in HICs, and this further reflects inequities in HPV vaccination access.^1^ Financial and programmatic barriers including high vaccine cost, lack of political will, vaccine logistics challenges, health workforce gaps, and global HPV vaccine supply constraints have been adduced as major reasons for inequitable national introduction of HPV vaccination especially in LMICs where vaccines are needed the most.^6,11,12^

The interventions of Gavi, The Vaccine Alliance—a global public-private partnership targeted at addressing major precursors of vaccine inequity such as high vaccine prices and vaccine delivery logistics have been hailed as a masterstroke toward improving global access to HPV vaccines especially in LMICs.^6,13^ At the heart of Gavi partnerships are its core members—WHO, UNICEF, World Bank, and Bill and Melinda Gates Foundation and other key development partners including governments, civil society organizations, vaccine manufacturers, research agencies, and other private sector players. Gavi achieved a historic agreement with vaccine manufacturers—GlaxoSmithKline and Merck to reduce the price of the bivalent (cervarix) and quadrivalent (gardasil) HPV vaccines which can cost more than $100 (USD) in HICs to less than $5 for Gavi-eligible countries where cervical cancer burden is the highest.^6,13^ Despite such historic agreements, it has been argued that the price is still high and not sustainable for LMICs including Nigeria within the context of pervasive poverty, overstretched health budgets, and competing needs for scarce resources.^4,14^

Beyond the agreement to make HPV vaccine affordable for target LMICs, Gavi provides significant funding and technical support for eligible countries that indicate interest to introduce HPV vaccines via two pathways—one for the national introduction of HPV vaccines and the other as a demonstration program.^6,13^ The national introduction pathway involves eligible countries to meet a Gross National Income per capita (GNIpc) of ≤$1,580 (USD), at least a 70% diphtheria-tetanus-pertussis third dose coverage, and have shown the capacity to deliver a multidose vaccine to at least 50% of 9- to 14-year-old girls.^6,14^ However, Gavi's funding model and reliance on national income to determine eligible countries for support have been criticized as exclusionary on the basis that the GNIpc metric may not reflect the true economic realities of LMICs.^11,14^ Such exclusion potentially translates to widening inequities when countries that bear the greatest burden cannot afford the negotiated vaccine price and are not eligible to access vital support to roll out life-saving HPV vaccines.

Gavi's demonstration program pathway is a 2-year project which allows countries the opportunity to gain experience in delivering HPV vaccines to a target population (9- to 14-year-old girls) not usually involved in routine immunization schedules and make decisions to either introduce HPV vaccine nationally or not. Precisely, country decisions on national introduction are expected to be informed by the key lessons provided by the demonstration program on issues such as vaccine costs and logistics, coverage, acceptability, and feasibility.^6^ Gavi covers the vaccine supply cost until the port of entry and provides further cash grant that supports about 80% of operational costs under the demonstration program. New Gavi guidelines released in 2016 now allow for countries yet to introduce HPV vaccines like Nigeria to apply directly for Gavi support to fund national or phased introductions rather than starting with a demonstration program from 2017 onwards.^11-13^ The phased introduction approach and availability of empirical evidence of what works for successful HPV vaccination implementation represent critical opportunities for LMICs including Nigeria yet to introduce HPV vaccines to leverage.

Two strategic documents, National Strategic Plan on Prevention and Control of Cervical Cancer in Nigeria (2017-2021)^15^ and National Cancer Control Plan (2018-2022),^16^ had set the roadmap for Nigeria's vision for cervical cancer prevention including specific plans and targets for HPV vaccination. For instance, the National Strategic Plan on Prevention and Control of Cervical Cancer in Nigeria (2017-2021) highlighted Nigeria's intention to leverage high-volume catchment areas toward delivering two doses of quadrivalent HPV vaccine to 4.5 million girls age 9-13 years over a 5-year period at a financial cost of $18 million (USD) ($3.98 per fully immunized girl). Similarly, the National Cancer Control Plan (2018-2022) boldly set an objective of attaining 90% HPV vaccine coverage for girls age 9-13 years in Nigeria by 2022. These ambitious goals outlined by both strategic documents were never achieved.^17^ Nevertheless, such documents could serve as the foundation for future national cervical cancer prevention plans especially HPV vaccine introduction.

During the national stakeholder's forum on the elimination of cervical cancer held in November 2020, Nigeria's health minister Osagie Ehanire in his keynote speech announced the country's plan to introduce HPV vaccines by 2021.^17^ This announcement was met with renewed optimism and expectations. However, more than 2 years later, HPV vaccination has not been rolled out in Nigeria. When the 2021 deadline was missed, Faisal Shuaib, executive director of the National Primary Health Care Development Agency (NPHCDA), made a similar announcement for a new date on July 14, 2022.^17^ Unsurprisingly, much skepticism exists about the likelihood of introducing HPV vaccination by the new date. Specifically, Shuaib announced that HPV vaccines for Nigerian girls (9-14 years) will be introduced into Nigeria's expanded immunization program by the third quarter of 2023.^18^ Concerns around the feasibility of implementation considering high vaccine cost which is the largest contributor to vaccine delivery costs and if Nigeria will get needed support including partnerships to roll out HPV vaccines in 2023 persist. However, to address these concerns, the NPHCDA executive director recently stated that the country had met the conditions for support from Gavi and obtained provisional approval for a phased HPV vaccine introduction and subsequently, provide the same as part of routine immunization after 2024.^18^

In some areas in Nigeria, HPV vaccines are sparsely provided in private and public health facilities at an average cost of $13 (USD) which is expensive considering the high poverty levels in the country and further impedes access for individuals who need them the most but cannot afford it.^19^ Although low awareness of HPV and HPV vaccines has been reported among parents of Nigerian girls as barriers to vaccinating their kids, their willingness to vaccinate their daughters is high.^20^ Similarly, evidence from a pilot school-based HPV vaccination program revealed the high acceptability of HPV vaccines among parents and adolescent girls in Nigeria.^21^ Such a high level of willingness and acceptability of HPV vaccines further strengthens the rationale for the national introduction of HPV vaccines in Nigeria.

Moving forward, if Nigeria is to make considerable progress in fully immunizing the estimated 15 million Nigerian girls age 9-14 years, the country must be deliberate in leveraging every opportunity that puts them in a good position to sustainably roll out HPV vaccines nationally. Strong political commitment to prioritize and drive needed actions for the introduction of HPV vaccines and sustaining the gains of such introduction in Nigeria is critical.^11,14^ The success of the HPV vaccine rollout in Rwanda since 2011 which put the country on a path toward the elimination of cervical cancer after high HPV vaccine coverage (92.23%) of the target population serves as a key example of what strong political commitment, government ownership, and leadership can accomplish.^22^

To meet global and national HPV vaccination targets, the significance of global partnerships that stimulate national action, address funding concerns including vaccine costs, and strengthen equitable vaccine supply access for countries with the highest cervical cancer burden has been emphasized.^1,14,22^ Hence, Nigeria must leverage collaborative partnerships and ensure coordination between government and relevant development partners including bilateral and multilateral organizations, vaccine manufacturers, private sector stakeholders, and community-based and civil society organizations in driving advocacy, policy, and program implementation efforts that creates access to HPV vaccination especially for the most disadvantaged girls. Again, Rwanda's partnership with Merck (a pharmaceutical company) that led to the latter agreeing to supply HPV vaccines for the first 3 years of national introduction at no cost to the country highlights the power of partnerships and provides lessons for Nigeria.^22^ Rwanda's agreement with Merck which made the country the first low-income country to introduce HPV vaccines also demonstrated how partnership can facilitate the achievement of shared goals. For instance, although Merck was able to show the feasibility of introducing HPV vaccines in LMICs, Rwanda made huge progress in protecting girls from cervical cancer through lifesaving HPV vaccines. Furthermore, Nigeria must also prioritize interministerial collaboration particularly involving the finance, health, and education ministries as part of the foundations for HPV vaccine rollout and improved HPV vaccination outcomes.^12^

Nigeria's intention to introduce HPV vaccines must rely on evidence-based and comprehensive introduction plans. These plans should recognize the peculiarities of the target population (girls age 9-14 years) through microplanning, clearly outline financing strategies and cost implications for introduction and sustainability including operational and logistics costs, and prioritize diverse vaccine delivery mechanisms including integrated approaches, community outreaches, and school-based vaccination programs. In addition, the plan must provide for health system strengthening including health workforce and infrastructure initiatives and include a robust communication and social mobilization approach underlined by meaningful community engagement that improves vaccine acceptability, increases population awareness, and translates willingness to vaccinate into action.^11,12,23^

As Nigeria prepares to roll out HPV vaccination, it is equally important to establish strong accountability mechanisms including robust monitoring and evaluation frameworks that highlight progress, identify multilevel challenges across the HPV vaccine introduction process, and proffer solutions to improve program operations and sustainability.
